# Lethal autonomous weapon systems and respect for human dignity

**DOI:** 10.3389/fdata.2022.999293

**Published:** 2022-09-09

**Authors:** Leonard Kahn

**Affiliations:** College of Arts and Sciences, Loyola University New Orleans, New Orleans, LA, United States

**Keywords:** military ethics, AI ethics, lethal autonomous weapon systems, artificial intelligence, applied ethics

## Abstract

Much of the literature concerning the ethics of lethal autonomous weapons systems (LAWS) has focused on the idea of human dignity. The lion's share of that literature has been devoted to arguing that the use of LAWS is inconsistent with human dignity, so their use should be prohibited. Call this position “Prohibitionism.” Prohibitionists face several major obstacles. First, the concept of human dignity is itself a source of contention and difficult to operationalize. Second, Prohibitionists have struggled to form a consensus about a property P such that (i) all and only instances of LAWS have P and (ii) P is always inconsistent with human dignity. Third, an absolute ban on the use of LAWS seems implausible when they can be used on a limited basis for a good cause. Nevertheless, my main purpose here is to outline an alternative to Prohibitionism and recognize some of its advantages. This alternative, which I will call “Restrictionism,” recognizes the basic intuition at the heart of Prohibitionism - namely, that the use of LAWS raises a concern about human dignity. Moreover, it understands this concern to be rooted in the idea that LAWS can make determinations without human involvement about whom to target for lethal action. However, Restrictionism differs from Prohibitionism in several ways. First, it stipulates a basic standard for respecting human dignity. This basic standard is met by an action in a just war if and only if the action conforms with the following requirements: (i) the action is militarily necessary, (ii) the action involves a distinction between combatants and non-combatants, (iii) noncombatants are not targeted for harm, and (iv) any and all incidental harm to non-combatants is minimized. In short, the use of LAWS meets the standard of basic respect for human dignity if and only if it acts in a way that is functionally isomorphic with how a responsible combatant would act. This approach leaves open the question of whether and under what conditions LAWS can meet the standard of basic respect for human dignity.

Much of the literature concerning the ethics of lethal autonomous weapons systems (or LAWS as they are usually called) has focused on the idea of human dignity, and the lion's share of that literature has been devoted to arguing that (1) the use of LAWS without meaningful human control violates human dignity, so (2) their use is morally prohibited and, therefore, (3) should be illegal.^1^Peter Asaro provides an admirably clear statement of this view when he writes,

As a matter of the preservation of human morality, dignity, justice, and law we cannot accept an automated system making the decision to take a human life. And we should respect this by prohibiting autonomous weapon systems (Asaro, 2012).^2^

## Call this view “Prohibitionism.”

There is much to be said in favor of Prohibitionism. It proceeds from intuitive premises, and it has clear policy implications that align well with general concerns about limiting the use of lethal violence in war. However, I take a different but related approach in this paper. In particular, I sketch an underexplored alternative to Prohibitionism and briefly offer considerations in favor of it. I conclude by suggesting some implications for the development of LAWS.

In order to understand this alternative view a little better, it will be helpful to take a step back and get a better look at the broader conceptual landscape. To that end, consider two claims that will be of central importance throughout my discussion:

- The Permissibility Claim: With respect to considerations of human dignity, it is morally *permissible* to use LAWS.
- The Requirement Claim: With respect to considerations of human dignity, it is morally *required* to use LAWS.^3^

There are a number of ways to mix and match evaluations of the Permissibility Claim and the Requirement Claim, but only two are important here. First, Prohibitionists are committed to the view that both the Permissibility Claim and the Requirement Claim are always false. Second, the alternative that I'll explore maintains that both the Permissibility Claim and the Requirement Claim are sometimes true and sometimes false, depending on context, particularly the level of technological development. I'll call this alternative “Weak Restrictionism.”

More about Weak Restrictionism in a moment. I owe the reader a few words about the idea of human dignity, though I can treat this topic only superficially in the space available to me. It is important to begin by acknowledging frequent complaints that human dignity is a “useless concept” (Macklin, 2003) or a “squishy, subjective notion, hardly up to the heavyweight moral demands assigned to it” (Pinker, 2008). We should recall that complaints of this kind are not new or unique to discussion of LAWS. Michael Rosen reminds us that Schopenhauer denounced talk of the “dignity of man” as “the shiboleth of all the perplexed and empty-headed moralists who conceal behind that imposing expression their lack of any real basis of morals, or, at any rate, of one that had meaning” (Schopenhauer, 1998; quoted in Rosen, 2012, p. 1).^4^

Of course, it's easy to caricature and then ridicule the idea of human dignity. This is a tempting mistake - but a mistake nonetheless. While there's no doubt both that the concept is not fully understood (Kateb, 2014) and that competing interpretations of it are often difficult to operationalize (Polonko and Lombardo, 2005), we can try to make some progress.

For the purposes of this paper, I understand human dignity as a status concept.^5^ A little more precisely, human dignity concerns the moral standing that human beings have in virtue of the intrinsically valuable features that characterize most mature members of our species. According to this way of thinking, human dignity can play the role of justifying international humanitarian law (Regan, 2019, p. 213) and international human rights law (Luban, 2015, p. 270). Actions and attitudes that treat human beings as if they lacked these valuable qualities are threats to human dignity since they treat us in ways that are humiliating.^6^ For now, let me note that human dignity so understood need not be based on “absolute, unconditional, and incomparable value or worth” (Parfit, 2011, p. 239). The view that human dignity has no rivals with regard to the value on which it is based—a view associated with Kant (Dean, 2006, p. 37)—is consistent with what I will say here, though it is not entailed by it. All that matters for the purposes of this paper is that the phrase “human dignity” denotes a *high* moral status that imposes significant moral restrictions on certain classes of actions and attitudes. This is not the place to enumerate these features or explain why they are valuable. Such features, while perhaps not unknowable, are certainly not easily known and require a separate line of inquiry (Barrett, 2013, p. 5). Rather, I will fall in line with a tradition of grounding to at least some degree human dignity in personal autonomy, our capacity to govern ourselves in light of our values, suitably understood (e.g., Oshana, 2016).

Now that we have a barebones account of human dignity let's work our way back to Prohibitionism and Weak Restrictionism by means of the Principle of Discrimination, which requires us both to distinguish between enemy combatants and non-combatants and to avoid targeting the latter for harm.^7^ Some Prohibitionists connect the idea that the use of LAWS is a violation of human dignity with the idea that their use transgresses against the Principle of Discrimination (Gubrud, 2014).

Their use transgresses against the Principle of Discrimination, on this line of thinking, for at least two reasons. First, the sensory systems of LAWS are incapable of distinguishing reliably between combatants and non-combatants, because there is no algorithm for determining whether someone is a combatant. Or, as Sharkey puts it, LAWS “do not have adequate sensory or vision processing systems for separating combatants from civilians, particularly in insurgent warfare, or for recognizing wounded or surrendering combatants” (2012, p. 288). Second, LAWS lack higher-order situational awareness that would promote the capacity to make this distinction (Sharkey, 2019, p. 76). These are, I acknowledge, just two complaints. One might add that LAWS are likely to have a hard time recognizing attempts to surrender. But this will do.

This, if true, shows that the use of LAWS could be a violation of the Principle of Discrimination. But a further argument would be necessary to show that this violation would also constitute an offense against human dignity. Satisfactory arguments of this sort can be harder to find, but a promising start can be found with the observation that actions that ignore the distinction between combatants and non-combatants thereby fail to recognize and treat appropriately the value of the personal autonomy of those who have chosen not to become combatants and who have, therefore, rendered themselves essentially defenseless against the use of armed force.^8^If we transgress against the Principle of Discrimination, then we treat humans as if they were non-humans who cannot make the choice to refrain from harming others by not engaging in military service and, in the process, rendering themselves vulnerable—indeed mortally vulnerable—to others.^9^In doing so, we blatantly ignore one of the characteristic features of our species—personal autonomy—that makes our lives intrinsically valuable.^10^

I think we should grant the Prohibitionist the claim that violations of the Principle of Discrimination are infringements of human dignity in more or less the way that I've just suggested. And I think we should also acknowledge that the use of LAWS can be a violation of the Principle of Discrimination, especially given their fairly crude level of development at the moment. Hence, it is at least sometimes the case that the Permissibility Claim is false. But even if we concede that the use of the fairly primitive LAWS now available would be a violation of the Principle of Discrimination, it does not follow that the same will be true of the use of future LAWS. Along those lines, consider a somewhat complicated scenario I will call

Case 1: Alfastan is fighting a just war against Betaville. Gamma, an officer in Alfastan's military, has the opportunity to capture Point Delta, which is necessary to Alfastan's effort to accomplish its just war aims. However, the only soldiers available to Gamma at the moment are in Company Epsilon. Gamma believes that these warfighters will violate the Principle of Discrimination if they are ordered to take Point Delta. Nevertheless, Gamma can instead use highly sophisticated LAWS to take Point Delta. These futuristic LAWS can distinguish between combatants and non-combatants with a high degree of accuracy, thereby vastly lowering the risk of a violation of the Principle of Discrimination.^11^

It seems to me that it is permissible on the basis of considerations of human dignity for Gamma to use their LAWS instead of the soldiers in Company Epsilon to take Point Delta. This fact weighs against understanding the Permissibility Claim as always false, as Prohibitionists maintain. Or, to put the matter a little differently, if my judgment is correct then it is sometimes permissible on the grounds of human dignity to use LAWS. Now consider

Case 2: This case is similar to Case 1 except that by taking Point Delta, Gamma can prevent large-scale assaults on the basic human dignity of a group of non-combatants, which Gamma is certain will otherwise occur as a result of the actions by their adversary.

Plausibly, on the basis of considerations of human dignity, it is not only permissible but required for Gamma to use their highly sophisticated LAWS instead of their soldiers to take Point Delta in Case 2. That is to say, one would not be permitted not to use LAWS in this situation. Why? Only by using these LAWS, which do not themselves violate the Principle of Discriminaiton, can Gamma prevent the large-scale assaults on the human dignity of many non-combatants. If Gamma is required to do so, then there are at least some situations in which the Requirement Claim is true, a fact which is consistent with Weak Restrictionism but not with Prohibitionism.

It might be objected that the two thought experiments I've offered rely too heavily on warfighters being incapable or unwilling to act on the Principle of Discriminaiton. So consider what I will call the maximalist extension of the Principle of Discrimination: If one can employ several means M_1_, M_2_, … M_n_, etc. to achieve an otherwise morally legitimate military objective, and one of the means, M_i_ is more likely than any of the other means to distinguish non-combatants from enemy combatants while not targeting the former for harm, then there is a pro tanto reason to use M_i_ rather than any other means.^12^ The reason to favor M_i_ is only prima facie since there might be countervailing reasons that favor another means. Nevertheless, if the maximalist extension of the Principle of Discrimination is correct,^13^ then there are more possible circumstances in which the use of LAWS is not only permissible but required by considerations of human dignity. Consider

Case 3: The case is similar to Case 2. However, Gamma can also use Company Zeta to take Point Delta (to avoid the widespread assaults on human dignity), where Company Zeta, unlike Company Epsilon, would be unlikely to violate the Principle of Discrimination in the process. However, Gamma could instead use a highly advanced form of LAWS to take Point Delta. This LAWS is even more likely than the members of Company Zeta to distinguish accurately between enemy combatants and non-combatants and to avoid targeting non-combatants for harm.

In the absence of any countervailing considerations, I think Gamma is required by considerations of human dignity to use LAWS in Case 3 since it will achieve the same end of avoiding assaults on human dignity and do so by employing a means that is less likely to be a violation of human dignity.

More generally, Case 3 points to a surprising conclusion. Even in circumstances where it is possible meet *human* standards of discrimination, the continued development of technology and artificial intelligence might make it the case that we are required nevertheless to use LAWS precisely because doing so is more reliably discriminate. Warfighters might “be all they can be” yet still not be enough to do what they are required to do. In such cases it might be that human moral virtue must take a back seat to inhuman technological excellence.

Let me turn now to another suggestion made by Prohibitionists about how the use of LAWS is a threat to human dignity. This suggestion is more difficult to articulate adequately than the previous one, and it has many critics. My aim here is not to demonstrate its truth; it is to show that, even if we conceded to Prohibitionists that LAWS are a threat to human dignity in this way, Weak Restrictionism is still a more plausible position than Restrictionism. Greg Reichberg and Henrik Syse get us off to a good start with regard to this suggestion when they propose the possibility that “To be killed by machine decision would debase warfare into mere slaughter, as though the enemy combatant were on a par with an animal killed on an automated conveyor belt” (Reichberg and Syse, 2021, p. 153).^14^ However, it might get slightly closer to the view of many Prohibitionists to say that the use of LAWS debases not warfare but rather the human dignity of those who are caught up in it.

In order to get a better grip on this slippery idea, imagine (and here I betray my roots as a full-time college administrator) that your university has just purchased a derelict building that it plans on tearing down and replacing with a new dormitory for the university's students. However, the derelict building is infested with vermin, who are carriers of a lethal disease. While it is possible to send in workers to eradicate the pests, it is also possible to send in an autonomous robot to do the job. All other things being equal, many of us would think that sending in the autonomous robot is no worse—and possibly much better—than sending in workers to do the same. The thought here is that when it comes to eliminating vermin, there is nothing wrong *per se* about allowing lethal determinations to be made by non-humans. But many of us balk when it comes to permitting these lethal determinations to be made by non-humans when it is *human* lives on the line. The thought continues that, even if the autonomous robot is just as accurate as a human being concerning who is to be targeted with lethal force, the use of the autonomous robot transforms an act of war into something akin to the slaughter of an unwanted rodent. The use of an autonomous robot, such as a LAWS, expresses, the thought concludes, an attitude of contempt toward humans that amounts to an assault on human dignity. Note that the point here is not that the deaths of those killed by LAWS would be more painful, more protracted, or more gruesome. It might or might not be any of these things, but these issues are orthogonal to the point at hand - namely, that the action expresses contempt for the combatants and their value as human beings.

Before commenting on this suggestion, I will need to make two further points about the nature of human dignity. First, it is possible to distinguish two distinct levels of human dignity. This distinction is easier to see when considering concrete examples of human dignity being violated. Begin with the plausible thought that being denied certain goods that are not central to the exercise of personal autonomy over the course of an entire life can count as a violation of one's human dignity. For example, suppose that your sexual orientation is not those of the majority of the people among whom you live.

Nevertheless, I think that there is a significant qualitative difference between violations of human dignity of this sort and violations associated with, for instance, torture (Luban, 2007, p. 162–204), rape (Nussbaum, 2009), and enslavement (Hörnle, 2012). In cases such as these, the contravention of human dignity is a thing apart from the cases of bigotry described above, loathsome though they are. I can offer no more than a promissory note here to explain this difference in greater detail, but I think it will do for our present purposes to distinguish between basic human dignity and non-basic human dignity. Violations of basic human dignity are those that have a significant negative effect on our most fundamental abilities as humans to live self-directed lives within the bounds of morality. To be sure, this distinction between basic and non-basic human dignity deserves more care and can be drawn with considerably more nuance than I have allowed myself here. Nevertheless, a rough-and-ready version of the distinction will be enough for the moment.

Here is the second point I want to make about human dignity. Addressing issues of basic human dignity is considerably more urgent than addressing issues of non-basic human dignity. Rather dogmatically, I will say that the two forms of human dignity have something approximating a lexical relationship with regard to their moral importance. Let me spell out in some detail what I mean by that claim. Consider an agent *A* who has in their power the ability by acting to affect a group of stakeholders *S*_1_ through *S*_n_. Imagine that if *A* can do either one of two actions, *a*_1_ or *a*_2_. And further imagine that if A does *a*_1_, then they will ensure that at least one of *S*_1_ through *S*_n_ does not suffer a violation of their basic human dignity. However, if *A* does *a*_2_, then *A* will not ensure that at least one of *S*_1_ through *S*_n_ does not suffer a violation of their basic human dignity, though they will bring it about that at least one of *S*_1_ through *S*_n_ does not suffer a violation of their non-basic human dignity. Let the Priority Hypothesis be that in these conditions *A* is required to do *a*_1_, even though doing so will allow for a violation of non-basic human dignity among the stakeholders.

Even in its current form, the Priority Hypothesis provides support for Weak Restrictionism. In order to see why this is the case, return to Case 1. Assume for the sake of argument that the use of Epsilon Company to take Point Delta does not express contempt for the human dignity of the combatants who would defend it but, consistent with the suggestion of the Prohibitionists, that the use of LAWS would do so. But recall from this thought experiment that the LAWS will also more reliably distinguish between enemy combatants and non-combatants and while avoiding targeting non-combatants for harm than the other options available to Gamma. I think it is plausible to say that disregarding the opportunity to safeguard non-combatants is most likely a violation of basic human dignity, while expressing contempt toward combatants is more credibly a violation of non-basic human dignity. An act that fails to be as discriminate as possible certainly has a significant negative effect on our most fundamental abilities as humans to live self-directed lives within the bounds of morality since it will, for that very reason, result in injuries and death to those who have chosen not to be combatants. However, an act that expresses contempt toward combatants will not, *per se*, have a negative impact of this kind. The combatants who are injured and killed by the LAWS will not be diminished as humans by the attitude expressed by the use of the LAWS. The expression itself will not prevent them developing and promoting the valuable properties that are characteristic of being human. But the Priority Hypothesis requires us to give greater priority to avoiding the violation of basic human dignity than the violation of non-basic human dignity.

To say all of this, of course, is not to endorse or to trivialize such expressions of contempt. If Prohibitionists are correct that the use of LAWS does express disdain for human dignity, then there will be many situations in which it is impermissible to use them. However, Weak Restrictionists accept this point; indeed, they insist on it. They differ from Prohibitionists in virtue of also accepting the claims that it is sometimes permissible and even required to use LAWS because of considerations of human dignity. And it appears that in Case 1, the use of LAWS is required even if we grant the point that their use involves a violation of non-basic human dignity. Indeed, that is just what Weak Restrictionists have in mind when they assert that both the Permissibility Claim and the Requirement Clam are sometimes true. One need not throw up one's hands about the possibility of dignified death in war to acknowledge this (Scharre, 2018, p. 288). Nor is it necessary to hold out the hope that “one can maintain dignity in the face of indignity” (Young, 2021, p. 173). Weak Restrictionists need only maintain considerations of human dignity sometimes favor the use of LAWS all things considered, even if they do not do so unambiguously because it is sometimes impossible to act in ways that have no negative consequences for human dignity. The use of LAWS would not be unique among military actions in being ethically ambiguous for this reason. Something like this might also be true when it comes to, for example, the ethics of military intelligence (Bailey and Galich, 2012, p. 86).

One of the conceits of this paper is that it is possible that there will be LAWS that are better than humans—perhaps far better than humans—with respect to distinguishing combatants from non-combatants in the battle space. Implicitly, I've assumed that LAWS can do many other things better than humans can to avoid violations of human dignity as well. These ideas are far from new (Arkin, 2010). However, they are also far from being true of our world as I write this sentence in July 2022. Currently, there are few if any contexts in which it would be reasonable to expect LAWS consistently to perform better than well-trained and conscientious human combatants with respect to discrimination. Moreover, it is impossible to say with certainty when (or perhaps even *if*) LAWS will become robust and competent enough to be deployed in combat without meaningful human control. So it's natural to wonder whether introducing the possibility of futuristic LAWS does anything to advance the conversation.

Let me conclude by explaining why all of this—I think—matters. If Prohibitionists are correct, then considerations of human dignity require us to halt research and development into LAWS. Since they cannot be used in a manner that is consistent with human dignity, they should not be developed, and all of our energy should go into banning their use. Recall from the beginning of this paper Peter Asaro's insistence that “we should respect this [i.e., human dignity] by prohibiting autonomous weapon systems.” But if Weak Restrictionists are correct, then considerations of human dignity actually require further research into and development of LAWS since there are possible circumstances in which human dignity demands their use. Furthermore, if Weak Restrictionists are right, then considerations of human dignity at least partially ought to set the agenda for how LAWS are to be developed. It also ought to inform any and all attempts to regulate the development and use of LAWS at the international level. So the implications of the two views are very different, and the fact that the LAWS I've used in thought experiments throughout this talk are not yet extant is no objection.^15^

## Data availability statement

The original contributions presented in the study are included in the article/supplementary material, further inquiries can be directed to the corresponding author/s.

## Author contributions

The author confirms being the sole contributor of this work and has approved it for publication.

## Conflict of interest

The author declares that the research was conducted in the absence of any commercial or financial relationships that could be construed as a potential conflict of interest.

## Publisher's note

All claims expressed in this article are solely those of the authors and do not necessarily represent those of their affiliated organizations, or those of the publisher, the editors and the reviewers. Any product that may be evaluated in this article, or claim that may be made by its manufacturer, is not guaranteed or endorsed by the publisher.

## Footnotes

^1^I will be concerned in this paper only with the use of LAWS *without* meaningful human control since their use with meaningful human control - i.e., without full autonomy - is less interesting and involves complications and qualifications that I cannot address here. In order to avoid tedium, I will drop the phrase “without meaningful human control” in the rest of this paper, but I take it as understood. On meaningful human control, see Santoni de Sio and Van den Hoven (2018).

^2^See also, e.g., Docherty (2014), Sparrow (2016), Heyns (2017), Rosert and Sauer (2019), Sharkey (2019), Sauer (2021), and Saxon (2021). I look at some arguments offered in favor of Prohibitionism later in this paper. For earlier criticism, see Birnbacher (2016).

^3^Obviously, the domain of agents affected by the Requirement Claim is limited to those who can use LAWS since “ought,” in some suitable sense, entails “can” (Griffin, 2010). I understand permitted and required in the standard way these terms are mutually defined in deontic logic. X is permitted to do F if and only if X is not required not to F, and X is required to do F if and only if X is not permitted not to do X (Føllesdal and Hilpinen, 1970; Rönnedal, 2010, p. 29).

^4^Rosen's characterization of Schopenhauer as the “the Ebeneezer Scrooge of nineteenth-century philosophy,” is too good to leave unmentioned.

^5^Luban (2007, p. 89) and Lysaker and Syse (2016, p. 117–118).

^6^On human dignity as non-humiliation, see Margalit (1996), Jaber (2000), Nussbaum (2009), Luban (2009), Killmister (2010), Sharkey (2014), Vorhaus (2015), Coghlan (2018), and Gisbertz (2018).

^7^The devil is in the details when it comes to the Principle of Discrimination, as is so often the case. See Nagel (1972), Walzer (1977, p. 160–175), McMahan (2009, p. 11–12), and Frowe (2015, p. 82–83). However, discussion of these details can be postponed until another time, and the flatfooted distinction I make here between combatants and noncombatants can be nuanced as necessary.

^8^The fact that non-combatants have chosen not to make themselves liable to attack does not entail that all combatants have chosen to do so. Whether all combatants are indeed liable to attack is not something that needs to be resolved in this paper. I am grateful to an anonymous referee for pushing me to clarify this point.

^9^Perhaps those who have been coerced into the role of combatant have a different status than those who have assumed the role in the absence of coercion. But the brevity of this paper requires painting with a broad brush.

^10^For an alternative approach to human dignity and the Principle of Discrimination, see Kasher (2014).

^11^An anonymous referee pointed out, reasonably enough, a similarity between Case 1 and some thought experiments in Strawser (2010). However, Strawser's focus is on neither autonomous weapons nor human dignity.

^12^To be sure if there are two means M_j_ and M_k_ that are equally likely than another of the other means to distinguish between enemy combatants and noncombatants while not to targeting the latter for harm, and no other means are more likely than these, than there is a pro tanto reason to use either M_j_ or M_k_.

^13^One way to vindicate the maximalist extension of the Principle of Discrimination is by means of the Doctrine of Double Intention. See Lee (2004) and Zohar (2007).

^14^See also, e.g., Goose and Wareham (2016), Leveringhaus (2018), and Rosert and Sauer (2019).

^15^I am very grateful to attendees of the 2022 McCain Conference at the US Naval Academy and the 2022 meeting of the International Society of Military Ethics at the University of Colorado, Colorado Spring as well as to two anonymous referees from their comments, questions, and criticisms. Readers of this paper who have spent the 2021–2022 academic year with me in the Stockdale Seminar will recognize the degree to which I am indebted to its members for shaping - and reshaping - my thinking on this topic. I am deeply grateful to them for this. I have attempted to register these debts in the parenthetical references and footnotes, though I doubt I have managed to do so in every case where I should have. Whatever flaws remain in the paper are my own.
